# High-dose intravenous immunoglobulin therapy for novel coronavirus disease 2019: a brief report of two cases

**DOI:** 10.1186/s42077-020-00089-4

**Published:** 2020-09-10

**Authors:** Cem Erdogan, Bahadir Ciftci, Deniz Kizilaslan, Mursel Ekinci, Gülsen İptec, Ali İhsan Gemici, Pelin Karaaslan

**Affiliations:** 1 Department of Anesthesiology and Reanimation, Mega Medipol University Hospital, Istanbul Medipol University, School of Medicine, Bagcilar, 34040 Istanbul, Turkey; 2 Department of Hematology, Istanbul Medipol University, School of Medicine, Istanbul, Turkey

To the Editor,

Novel coronavirus disease 2019 (2019-nCoV) is a global pandemic which threatens all over the world. The first cases were seen in Wuhan, China, in December 2019 (Guo et al. 2020; Chen et al. 2020). It has spread rapidly, and now, there are more than 2.3 million reported cases and 160,000 deaths worldwide. 2019-nCoV may cause multi-system infections especially respiratory infections such as severe acute respiratory syndrome (SARS) and Middle East respiratory syndrome (MERS) (Guo et al. 2020; Chen et al. 2020; Sanders et al. 2020). In some cases, infections may be mild (only cough or fever, etc.); however, in some cases, infections may be severe (acute respiratory failure with diffuse pneumonia). In severe cases, there is an exaggerated immune response with low peripheral lymphocyte levels and high cytokine levels. This mechanism of pathogenesis may be responsible for the multiple organ failures (Cao et al. 2019; Lin et al. 2020). In this early stage of the 2019-nCoV, the infection may be treated successfully with immunomodulation (Cao et al. 2019). So, the immunomodulation options like intravenous immunoglobulin should be kept in mind. The intravenous immunoglobulin (IVIg) has been used as a replacement therapy in some immunodeficiency syndromes (Ballow 2008; De Ranieri 2017). IVIg plays an immunomodulatory and anti-inflammatory role in such cases like 2019-nCoV (Cao et al. 2019; Lin et al. 2020). Therefore, IVIg may be used as a supportive immunomodulatory drug as a part of the treatment of 2019-nCoV. We want to share our high-dose IVIg treatment experiences in two 2019-nCoV patients who recovered from the infection in our tertiary university hospital intensive care unit (ICU).

Written informed consents were obtained from the patients for this report. The first patient was a 35 years old man. He was diagnosed with 2019-nCoV with specific molecular test (polymerase chain reaction (PCR)) and thorax tomography. He had psoriasis as a comorbidity and received immunosuppressive treatment for 2 years. He was observed in our clinic for 3 days with a 2019-nCoV diagnosis. We admitted him to our ICU on the 4th day. He had tachypnea (respiratory rate 45/min) and dyspnea at ICU admission. His peripheral oxygen saturation was 75% with 10 l/min oxygen flow. He had received favipiravir and hydroxychloroquine treatment. In addition, we supported his respiration with non-invasive mechanical ventilation. However, his symptoms did not decrease and he was unstable. So, we started him high-dose IVIg (hospital day 6) at 1 g/kg for 2 days (totally 180 g for 2 days). On the second day of the IVIg treatment, the patient’s clinical status improved. He became afebrile, and there was no need of non-invasive mechanical ventilation. No adverse effect was reported. His oxygen saturation level was 95–97% with no oxygen support. Two consecutive PCR tests were negative. He was discharged from the ICU.

The second patient was a 76-year-old woman. She was diagnosed with 2019-nCoV with specific molecular test (polymerase chain reaction (PCR)) and thorax tomography. She had chronic myelocytic leukemia (CML) as a comorbidity for 2 years and received IVIG treatment routinely for CML (10 g sc for 15 days period). She was observed in our clinic for 2 days with a 2019-nCoV diagnosis. We admitted her to our ICU on the 3rd day. She had tachypnea (respiratory rate 38/min) and dyspnea at ICU admission. Her peripheral oxygen saturation was 65% with 10 l/min oxygen flow. She had received favipiravir and hydroxychloroquine treatment. We supported her respiration with non-invasive mechanical ventilation. However, her symptoms did not decrease, she was unstable, and we intubated her. So, we started high-dose IVIg (hospital day 5) at 1 g/kg for 2 days (in total, 180 g for 2 days). On the first day of the IVIg treatment, the patient’s clinical status improved. She became afebrile. After 6 days of IVIg treatment, she was extubated. There was no adverse event. Her oxygen saturation level was 96–98% with no oxygen support. Two consecutive PCR tests were negative. She was discharged from the ICU.

## Discussion

The aim of IVIg therapy is to provide antibodies against infections. IgG preparations are derived from a plasma pool of approximately 10,000 donors. These preparations contain mainly IgG (95–99%). It contains a large number of bioactive molecules. IVIg may be indicated for several conditions such as replacement therapy, anti-inflammatory treatment, and immunomodulatory therapy in different situations (Cao et al. 2019; Ballow 2008; De Ranieri 2017). High-dose IVIg may be used as an effective and safe immune modulator in 2019-nCoV infection.

In conclusion, IVIg may be performed in 2019-nCoV infection as a part of the main treatment for immunomodulation. However, further studies are needed to understand the efficacy of this strategy.

## Data Availability

Not applicable

## Abbreviations

2019-nCoV: Novel coronavirus disease 2019
SARS: Severe acute respiratory syndrome
MERS: Middle East respiratory syndrome
IVIg: Intravenous immunoglobulin
ICU: Intensive care unit
PCR: Polymerase chain reaction
CML: Chronic myelocytic leukemia
